# Pediatric Primary Diffuse Leptomeningeal Melanomatosis: A Case Report and Literature Review

**DOI:** 10.7759/cureus.105377

**Published:** 2026-03-17

**Authors:** Emily Hanus, Danielle Cunningham

**Affiliations:** 1 Department of Radiation Oncology, University of Kansas Medical Center, Kansas City, USA; 2 Department of Radiation Oncology, University of Kansas Cancer Center, Kansas City, USA

**Keywords:** leptomeningeal disease (lmd), pediatric cns melanoma, pediatric neuron-oncology, primary diffuse leptomeningeal melanoma, rare pediatric cancer

## Abstract

Primary diffuse leptomeningeal melanomatosis (PDLM) is an exceptionally rare and aggressive pediatric central nervous system (CNS) malignancy that often presents with nonspecific symptoms, leading to delayed diagnosis. We report a seven-year-old boy who initially presented with recurrent focal neurologic episodes and headaches that were attributed to migraine before progressive visual changes and signs of elevated intracranial pressure (ICP) prompted further evaluation. Cerebrospinal fluid (CSF) cytology and meningeal biopsy confirmed PDLM with diffuse leptomeningeal involvement. Despite rapid neurologic decline after initiation of immunotherapy, urgent cranial and later spinal radiotherapy resulted in meaningful functional recovery, allowing temporary stabilization, return to school, and preservation of quality of life. Disease progression ultimately occurred, and he died 16 months after symptom onset. This case highlights the diagnostic challenges of PDLM and highlights the potential role of radiotherapy in providing neurologic improvement and symptomatic benefit, even in the setting of limited overall survival.

## Introduction

Primary diffuse leptomeningeal melanomatosis (PDLM) is an extremely rare and aggressive central nervous system (CNS) malignancy characterized by diffuse infiltration of atypical melanocytes throughout the leptomeninges [1,2]. The leptomeninges, composed of pia and arachnoid mater, envelop the brain and spinal cord and are populated by melanocytes derived from neural crest cells. During embryogenesis, these melanocytes migrate along the developing neural tube and localize within the leptomeninges, particularly at the skull base and ventral spinal cord. Malignant transformation of these resident melanocytes can lead to widespread dissemination without formation of a discrete parenchymal mass, resulting in the diffuse characteristic pattern of PDLM [3].

Diffuse meningeal infiltration disrupts the normal cerebrospinal fluid (CSF) dynamics. Progressive meningeal thickening impairs CSF absorption at the arachnoid granulations and obstructs subarachnoid flow pathways, frequently resulting in hydrocephalus and elevated intracranial pressure (ICP) [1]. These pathophysiologic changes underlie the common clinical presentations of headache, nausea, vomiting, papilledema, seizures, and progressive neurologic decline observed in cases.

Under the 2021 World Health Organization classification of CNS tumors, primary CNS melanocytic tumors are categorized into circumscribed lesions (melanocytoma and primary malignant melanoma) and diffuse lesions (melanomatosis and melanocytosis), with PDLM representing the most aggressive diffuse variant. [4]. Diffuse melanomatosis is distinguished from benign melanocytosis by cytologic atypia, increased mitotic activity, and invasive growth. Unlike circumscribed primary malignant melanoma, PDLM lacks a well-defined mass and instead demonstrates extensive leptomeningeal involvement [3].

Primary melanocytic tumors of the CNS account for 1% of all melanomas and only 0.05% of primary brain tumors, and PDLM is particularly rare in the pediatric population [5]. The disease may occur sporadically or in association with neurocutaneous syndromes such as neurocutaneous melanosis, Sturge-Weber syndrome, or neurofibromatosis type 1 [5].

Establishing the diagnosis of primary CNS melanocytic tumors requires exclusion of extracranial melanoma, as metastatic melanoma to the leptomeninges is more common. Diagnostic criteria include (1) absence of melanoma outside the CNS on comprehensive dermatologic, ophthalmology, and systemic evaluation, (2) absence of other primary sites at staging workup or autopsy, and (3) histopathological confirmation demonstrating melanocytic neoplasm [6]. Immunohistochemical staining typically demonstrates positivity for melanocytic markers such as S100, HMB-45, and Melan-A. Neuroimaging often reveals diffuse leptomeningeal enhancement without a dominant parenchymal mass, while CSF analysis may show elevated protein or malignant cells but is frequently nondiagnostic.

Given its rarity, there are no standardized diagnostic or treatment guidelines. Treatment strategies have been directed by the knowledge of leptomeningeal metastasis originating from extracranial malignant melanoma and have included combinations of chemotherapy, immunotherapy, and radiation therapy [7,8]. Despite multimodal treatment approaches, the prognosis remains poor. Here, we present a pediatric case of PDLM as well as a review of the current literature to highlight diagnostic and therapeutic considerations. This article was previously presented as a meeting abstract at the 2026 American College of Radiation Oncology Conference on February 6, 2026.

## Case presentation

A seven-year-old boy with no significant past medical history presented with three months of recurrent headaches accompanied by right-sided hemiplegia, aphasia, and vision changes. The symptoms initially developed following a minor head trauma. Imaging at the time was unremarkable, and his symptoms resolved spontaneously. On physical examination, the patient was at his neurological baseline, and no abnormalities were noted. He was diagnosed with hemiplegic migraines with a plan for outpatient neurology follow-up.

**Figure 1:**
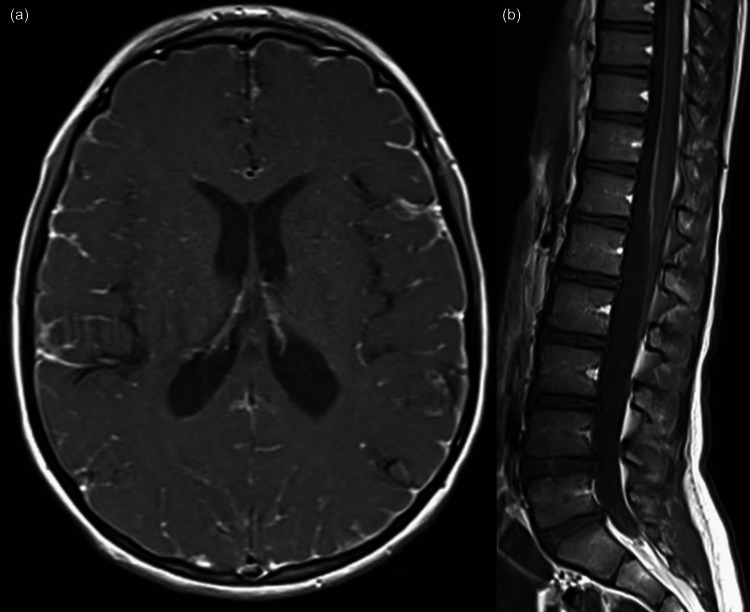
(a) MRI brain showing enhancement of leptomeninges and nonspecific signal changes in left frontal periventricular white matter. (b) MRI spine showing enhancement and focal thickening dorsal to the spinal cord at lower thoracic levels

Two weeks later, he returned with new-onset photophobia and vision changes. Ophthalmologic evaluation revealed bilateral papilledema. Lumbar puncture demonstrated a high opening pressure (too high to measure) and low glucose (29 mg/dL), and CSF cell counts revealed ~75% highly atypical cells. MRI of his brain and spine showed signal changes in cerebral sulci and leptomeningeal enhancement with differential diagnoses including meningitis, granulomatosis, inflammatory process, and malignancy (Figure 1). Biopsy of the dura and cortex revealed diffuse meningeal melanocytic neoplasm staining positive for Melan-A and HMB-45, most consistent with diffuse meningeal melanomatosis. Immunohistochemistry was negative for BRAF V600E. Positron emission tomography/computed tomography (PET/CT) scan showed no evidence of hypermetabolic neoplastic process outside of the craniospinal axis.

**Figure 2:**
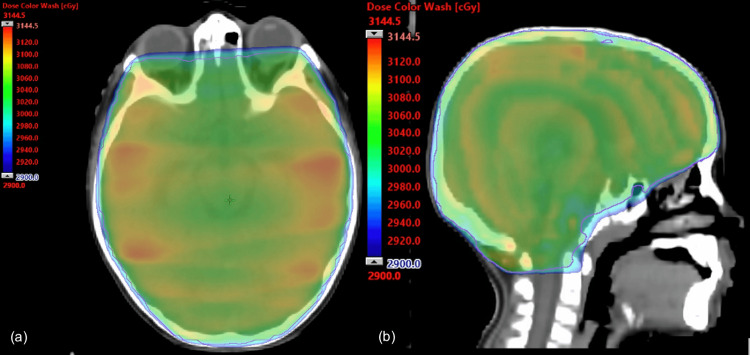
Whole-brain radiotherapy treatment plan using 3DCRT with 30 Gy delivered in 10 fractions 3DCRT: three-dimensional conformal radiation therapy

The patient was started on combination immunotherapy with nivolumab (1 mg/kg) and ipilimumab (3 mg/kg) every three weeks for four cycles [9]. Ten minutes into the infusion, he developed sudden dense paralysis and aphasia and was admitted to the pediatric intensive care unit. It was believed to be an acute infusion reaction. Symptoms were not due to increased ICP, and there was no hemorrhage shown on CT imaging. Continuous EEG did not show seizure activity. Within 48 hours, he regained a partial motor function but remained unable to walk or speak. Due to the rapid progression of symptoms as well as progression of leptomeningeal plaques on imaging, urgent whole-brain radiotherapy was recommended. At the time of the telehealth initial consult, his neurological status was an Eastern Cooperative Oncology Group (ECOG) performance status of 3-4. The patient underwent CT planning. He completed urgent whole-brain radiotherapy with three-dimensional conformal radiation therapy (3DCRT) of 30 Gy in 10 fractions (Figure 2). Prescription was decided due to the urgent neurologic deterioration for palliation. A three-point mask was used to immobilize the head without the need for sedation. He regained the ability to speak and walk within one week of treatment completion. His neurological status improved to ECOG 1. 

**Figure 3:**
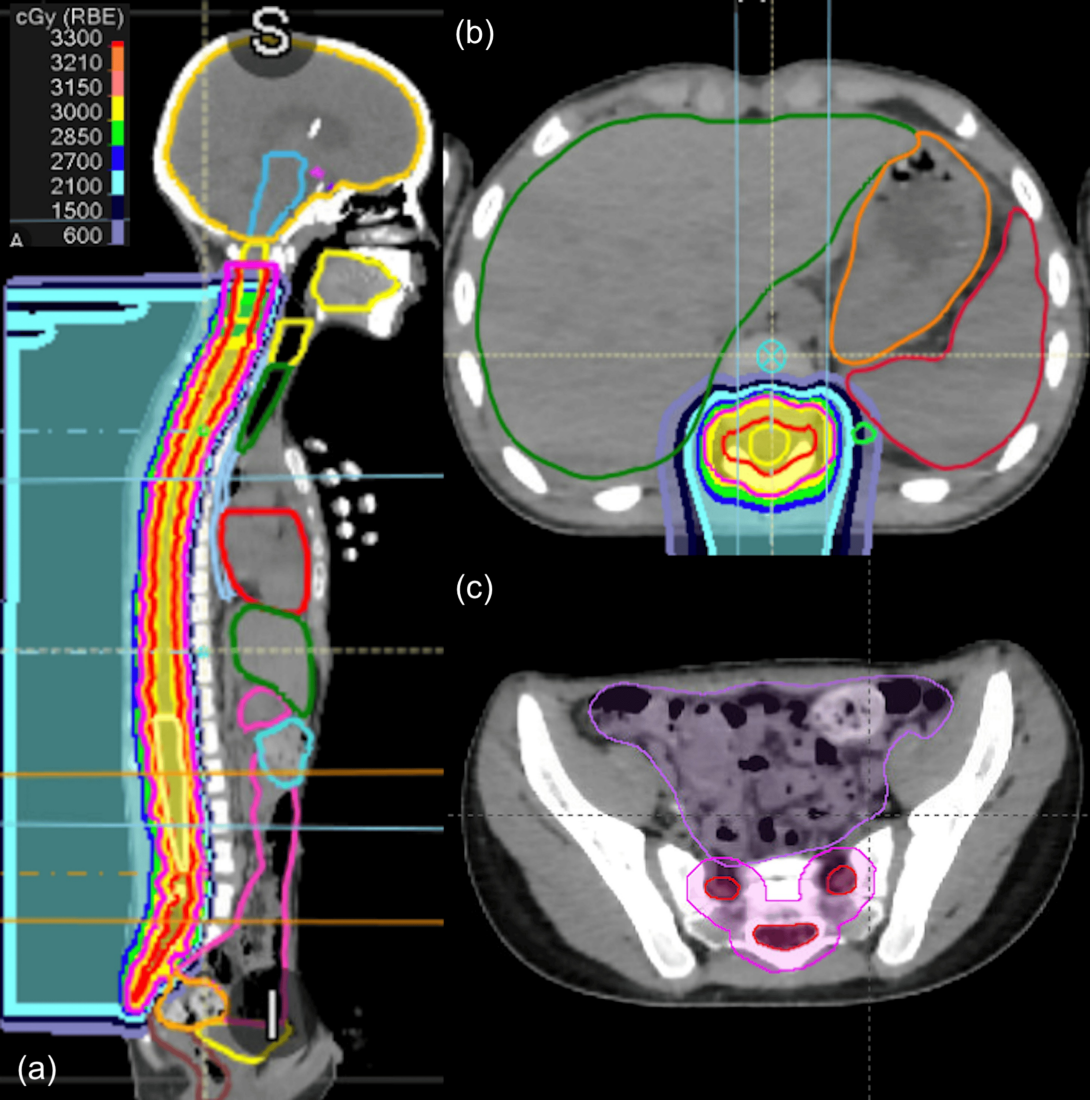
Urgent intensity-modulated proton radiotherapy of the spine with 30 Gy delivered in 10 fractions. Mean dose to the heart, esophagus, liver, small bowel, and large bowel was <1 Gy

One month into treatment, the patient presented with progressive lower extremity weakness, inability to walk, and loss of bowel and bladder control. His neurologic status was ECOG 3. MRI spine demonstrated malignant cauda equina syndrome. He completed 30 Gy in 10 fractions of whole-spine intensity-modulated proton radiotherapy (Figure 3). He was immobilized with a three-point head mask and Vac-Lok. Mean dose to the heart, esophagus, liver, small bowel, and large bowel was <1 Gy. One week after completion of radiotherapy, he gained full recovery of ambulation, and he gained the ability to hop and return to school within four weeks. 

Three months after completion of radiation therapy and four cycles of ipilimumab and nivolumab, MRI showed new involvement of the optic chiasm and optic nerve sheaths, but without evidence of spinal cord involvement. Repeat CSF remained positive for malignant melanocytic cells. His symptoms improved, and the patient was transitioned to monthly intravenous nivolumab monotherapy. The family elected to pause treatment after one dose. Approximately eight months after initiation of therapy, he returned with symptoms of elevated ICP. Imaging showed persistent leptomeningeal involvement, and CSF studies showed the presence of malignant cells. He began intrathecal nivolumab (20 mg) with concurrent IV nivolumab, as it has shown a positive outcome in adult patients [10,11]. He was on this combination therapy for a total of nine months. Nine months after radiation completion, he presented with seizures and was admitted to the PICU for status epilepticus requiring intubation and burst suppression. He recovered and was discharged home on antiepileptic medication.

**Figure 4:**
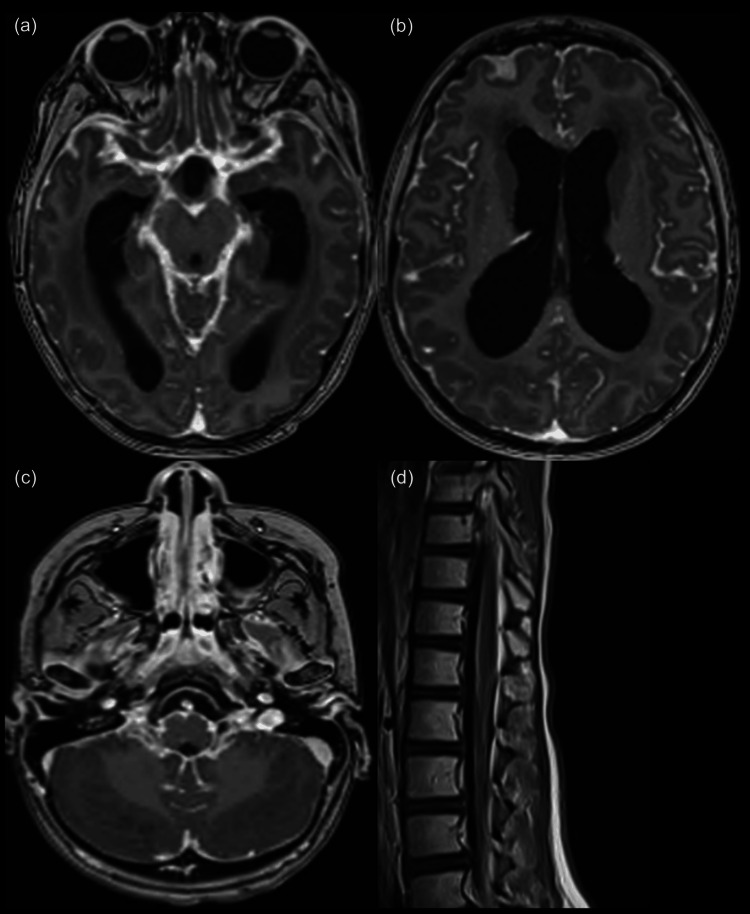
(a-d) MRI brain and spine showing extensively progressive disease both intracranial and intraspinal with stable ventriculomegaly

One year after completion of radiotherapy, the patient was still able to run and jump, though he had intermittent confusion. MRI demonstrated progressive leptomeningeal disease in the brain, filling the sulci and skull base with mild progression of spinal leptomeningeal disease (Figure 4). At this time, systemic therapy was stopped for lack of benefit. He later developed right-sided hemiparesis, aphasia, and vomiting, consistent with further progression. EEG was negative for seizures. He transitioned to hospice care and passed away five days later, 16 months after diagnosis.

## Discussion

To better contextualize our findings, we performed a comprehensive review of previously reported pediatric cases of PDLM, identifying 21 cases summarized in Table 1. PDLM remains one of the most aggressive and diagnostically challenging primary CNS malignancies in children. Our case reflects several hallmark features described in prior reports, including an initially nonspecific presentation, diagnostic delay, rapid neurologic decline, and poor overall prognosis. Importantly, it also demonstrates meaningful neurologic recovery following radiotherapy, highlighting the potential palliative and functional benefit of aggressive symptom-directed treatment.

**Table 1:** Reported pediatric cases of primary diffuse leptomeningeal melanomatosis CN: cranial nerve; CSF: cerebrospinal fluid; CSI: craniospinal irradiation; EVD: external ventricular drain; IHC: immunohistochemistry; LOC: loss of consciousness; NR: not reported; VP: ventriculoperitoneal; WBRT: whole-brain radiotherapy; FLAIR: fluid-attenuated inversion recovery

Author (year)	Age/sex	Key presentation	Imaging findings	CSF findings	Pathology/IHC/mutation	Treatment	Survival (months)
Nicolaides (1995) [18]	5M	Headache, vomiting, fever, CN palsy	CT: diffuse meningeal enhancement	Atypical multinucleated cells	Melanocytic infiltration of meninges with melanin pigment; IHC: S100+, HMB-45+: mutation testing: NR	Antituberculous therapy, steroids, chemotherapy, VP shunt	NR
Monsó (1996) [19]	6M	Intracranial hypertension, polyneuropathy	MRI: focal right thalamic lesion	↑ protein, ↓ glucose, no malignant cells	Autopsy-confirmed leptomeningeal melanomatosis; IHC: NR; mutation testing: NR	Steroids	NR
Makin (1999) [20]	5M	Headache, vomiting, LOC	CT: enhancing temporo-occipital lesion	↓ glucose, ↑ protein, acellular	Malignant melanocytic meningeal infiltration; IHC: vimentin+, S100+, HMB-45+; mutation testing: NR	Antitubercular therapy, chemotherapy, EVD	6
López-Castilla (2001) [21]	7M	Headache, vomiting, ataxia, dysarthria, LOC	MRI: diffuse subarachnoid hyperintensity, nodular extra-axial lesions with mass effect	Initially normal; later pleocytosis	Autopsy: diffuse malignant melanoma involving the subarachnoid space; IHC: NR; mutation testing: NR	Antiseizure therapy	1
Brunsvig (2011) [22]	10F	Seizures, vision changes, migraines, vomiting	MRI: parietotemporal and thalamic lesions, hydrocephalus	↑ protein, oligoclonal bands; atypical melanocytes on repeat	Leptomeningeal melanocytic infiltration; IHC: HMB-45+, Melan-A+; mutation testing: NR	EVD, antiseizure therapy	9
Lee (2013) [23]	17M	Headache, vomiting	MRI: diffuse right hemispheric leptomeningeal thickening	↓protein	Pleomorphic melanocytic cells with melanin pigment; IHC: S100+, HMB-45+; mutation testing: NR	WBRT, chemotherapy	NR
Szathmari (2016) [13]	5F	Headache, vomiting, seizures	MRI: leptomeningeal enhancement	Negative	Epithelioid melanocytic tumor; IHC: HMB-45+, Melan-A+; mutation testing: NR	Surgery, chemotherapy, VP shunt	11
Angelino (2016) [24]	2F	Vomiting, strabismus	MRI: diffuse brainstem and spinal leptomeningeal enhancement	Initially negative; malignant cells on repeat	Pleomorphic melanocytic cells; IHC: Melan-A+; mutation testing: NRAS Q61K + (postmortem)	Chemotherapy, radiotherapy	11
Xu (2020) [1]	13M	Headache, vomiting	MRI: left temporal hyperintense lesion	NR	Melanocytic tumor with melanin; IHC: Melan-A+, HMB-45+; mutation testing: NR	Surgical resection	5
Baumgartner (2021) [7]	14M	Headache, vomiting	MRI: diffuse craniospinal leptomeningeal enhancement; PET avidity in left parieto-occipital lobe	↑ protein, ↑ lactate, ↓ glucose	Pigmented perivascular tumor cells; IHC: HMB-45+, PD-L1+; mutation testing: NRAS Q61R +	Chemotherapy, targeted therapy, immunotherapy, EVD	7
Pezzullo (2021) [12]-case 1	16M	Headache	MRI: frontal subarachnoid FLAIR hyperintensity and dural thickening	NR	Pleomorphic pigmented melanocytic cells; IHC: HMB-45+, S100+, Melan-A+; mutation testing: NR	Chemotherapy	5
Pezzullo (2021) [12]-case 2	14M	Headache, vomiting	MRI: diffuse corticoleptomeningeal and spinal cord enhancement	NR	High-grade melanocytic tumor; IHC: HMB-45+, S100+, high Ki-67; mutation testing: NR	EVD, chemotherapy, WBRT + spine RT	5
Pezzullo (2021) [12]-case 3	15M	Headache, visual symptoms, CN VI palsy	MRI: extensive craniospinal leptomeningeal disease, mild hydrocephalus	NR	Pleomorphic melanocytic cells, scant pigment; IHC: NR; mutation testing: NR	VP shunt	8
Tavana Rad (2021) [25]	14F	Headache, diplopia, weakness	MRI: diffuse leptomeningeal FLAIR hyperintensity	Dry tap	Pigmented pleomorphic melanocytic tumor; IHC: NR; mutation testing: NR	Laminectomy	6
Yamauhci (2022) [15]	14M	Seizures, focal weakness	MRI: frontoparietal leptomeningeal enhancement extending into cerebral sulci	Atypical mononuclear cells	Melanocytic meningeal invasion; IHC: HMB-45+, high Ki-67; mutation testing: BRAFV600E -	WBRT (30 Gy in 15fx), immunotherapy, chemotherapy	20
Marquez (2023) [26]	14F	Headache, vomiting, photophobia	MRI: interhemispheric leptomeningeal enhancement	Disialoganglioside CD2+	Melanocytic neoplasm with brown pigment; IHC: HMB-45+, variable Ki-67; mutation testing: NR	Palliative care, VP shunt	4
Shahab (2024) [16]	3M	Vomiting, ataxia, headache	MRI: posterior fossa mass with diffuse craniospinal enhancement	Malignant cells present	High-grade melanocytic tumor; IHC: HMB-45+, Melan-A+, S100+; mutation testing: NRAS Q16R+, BRAFVE1-	Proton CSI (30 Gy in 10 fx) + boost 96Gy in 2 fx), immunotherapy	7
Sim (2024) [27]	12M	Fever, seizures, weight loss	MRI: diffuse craniospinal leptomeningeal thickening	NR	Melanocytic neoplasm; IHC: HMB-45+, Melan-A+, PRAME+; mutation testing: NRAS missense variant, BRAFV600E-	VP shunt, palliative care	NR
Kumar (2024) [8]	12M	Fever, vomiting, headache, seizures	MRI: suprasellar mass with craniospinal involvement	Negative	Pigmented melanocytic tumor; IHC: HMB-45+, Melan-A+; mutation testing: declined	VP shunt, steroids	4
Sareh (2025) [28]	10M	Headache, visual changes, hemiparesis	MRI: diffuse neuraxial leptomeningeal enhancement	Atypical mononuclear cells	Mature melanocytes without overt malignancy; IHC: S100, NSE, SOX10+; mutation testing: NRAS Q16K-	VP shunt, laminectomy	8
This Case	7M	Headache, hemiplegia, aphasia, vision changes	MRI: diffuse cerebral and spinal leptomeningeal enhancement	Atypical cells, ↓ glucose	Diffuse melanocytic neoplasm; IHC: HMB-45+, Melan-A+; mutation testing: BRAF V600E-	Immunotherapy, WBRT (30 Gy in 10 fx), proton CSI (30 Gy in 10 fx), VP Shunt	16

Consistent with prior pediatric cases, our patient initially presented with symptoms attributable to increased ICP and focal neurologic deficits. In our literature review, almost every patient presented with signs of increased ICP, most commonly headache, nausea, or vomiting, suggesting that impaired CSF circulation due to diffuse leptomeningeal infiltration represents a central pathophysiologic feature of PDLM. Eleven patients required an intervention to reduce ICP, including ventriculoperitoneal shunt placement or external ventricular drainage. Additional presenting symptoms included seizures (n = 5), cranial nerve palsies (n = 10), and loss of consciousness (n = 3) (Table 1). These nonspecific features often lead to diagnostic delays, as PDLM may initially mimic meningitis, subarachnoid hemorrhage, or inflammatory disorders. As in several previously reported cases, initial imaging in our patient was unrevealing, emphasizing the importance of repeat evaluation when neurologic symptoms persist or evolve. 

The diagnostic workup for PDLM requires a multimodal approach. On CT imaging, PDLM appears as hyperdensities within brain sulci, often mimicking subarachnoid hemorrhage [12]. MRI studies reveal diffuse leptomeningeal enhancement and may show hyperintensity signals on T1-weighted images or low T2 signal intensities due to the paramagnetic properties of melanin; however, these findings are not specific [13]. Systemic melanoma must be excluded for the diagnosis of PDLM, necessitating comprehensive dermatologic and ophthalmologic exams as well as whole-body PET/CT to rule out extracranial primary disease [14]. Given the limitations, CSF cytologic analysis is an important component of the diagnostic workup. In our review, malignant or atypical cells were found in 12 patients, including cases that required repeat lumbar punctures for confirmation. Additional CSF findings include elevated protein, due to leaking out from the disrupted blood-brain barrier and protein production by tumor cells, and low glucose due to increased metabolism of tumor cells [7]. Definitive diagnosis ultimately relies on meningeal biopsy with immunohistochemical staining for S-100, HMB-45, and Melan-A supporting melanocytic origin. Several cases reported a high Ki67 level, indicating aggressive tumor activity. Notably, NRAS mutations were identified in four patients, suggesting a potential molecular target for therapy. 

There are currently no established management guidelines for PDLM. Management strategies in the literature have varied widely and include supportive measures, systemic chemotherapy, immunotherapy, and radiotherapy. In our review, 11 patients received cancer-directed treatment, while 10 patients underwent symptomatic care or were unable to initiate therapy due to the rapid disease progression. Supportive measures included VP shunts to relieve hydrocephalus and anticonvulsant medications for seizure prophylaxis. Among those treated, 11 received chemotherapy or immunotherapy, and six underwent radiation therapy. The longest survival was reported as 20 months from diagnosis in a 14-year-old boy who was treated with whole-brain radiotherapy followed by immunotherapy [15]. Across previously reported cases of PDLM with available outcome data, the median survival was seven months (n = 17; IQR: 5-10 months; range 1-20 months). Most reported cases demonstrated survival of less than one year following diagnosis, highlighting the aggressive nature of PDLM and the need for more effective treatment strategies.

Radiotherapy may provide meaningful neurologic symptom control in leptomeningeal or metastatic CNS disease by reducing tumor burden, decreasing meningeal inflammation, and alleviating mass effect or CSF flow obstruction. In pediatric patients with diffuse neuraxis involvement, treatment approaches often prioritize rapid symptom relief while minimizing long-term toxicity, which may guide the selection of palliative whole-brain doses and targeted spinal irradiation. Notably, our patient experienced substantial neurologic recovery following whole-brain radiotherapy and subsequent spinal proton therapy. Restoration of speech, ambulation, and continence allowed meaningful preservation of quality of life and return to school for several months. Although prior reports have described transient neurologic improvement following radiotherapy, the degree and reproducibility of functional recovery observed in our patient are significant. [16]. The use of proton therapy for spinal irradiation also offers potential advantages in pediatric patients by improving dose conformality and reducing radiation exposure to surrounding normal tissues, including the heart, lungs, and abdominal organs, which may be particularly important when treating large portions of the neuraxis [17]. The survival of 16 months in our patient exceeded the reported median and approached the longest survivals documented in the pediatric literature. Our case supports the role of radiotherapy for symptomatic and functional benefit even in the context of diffuse leptomeningeal involvement. 

Systemic immunotherapy was also incorporated into our patient's treatment, reflecting evolving strategies extrapolated from cutaneous melanoma management. However, the benefit of immunotherapy in PDLM remains unclear, and molecular profiling may help guide future therapeutic decisions. 

This study is limited by the rarity of PDLM and reliance on case reports with heterogeneous treatment strategies and incomplete survival data. Nevertheless, aggregation of published cases provides valuable insight into clinical patterns and outcomes in this exceptionally rare disease. 

## Conclusions

PDLM is a rare and aggressive pediatric central nervous system malignancy that often presents with nonspecific symptoms, resulting in delayed diagnosis. The presented case highlights the diagnostic and therapeutic challenges associated with this disease. While available treatments are largely palliative, this case underscores the critical role of radiotherapy in achieving rapid neurologic improvement, preserving functional status, and maintaining quality of life. Radiotherapy should be considered an important component of multidisciplinary management for symptomatic disease and functional benefit, even in the setting of limited overall survival. Further collaborative research is needed to better define the optimal integration of radiotherapy with systemic therapies and to develop more effective treatment strategies for PDLM.
